# Control of Viral Aerosol Dispersion During Simulated Dental Procedures

**DOI:** 10.1016/j.identj.2025.103963

**Published:** 2025-10-23

**Authors:** Edgar O. Beltrán, James R. Allison, Nicholas S. Jakubovics, Jaime E. Castellanos, Richard Holliday, Myriam L. Velandia-Romero, Eliana P. Calvo, Manuel Forero, Stefania Martignon

**Affiliations:** a UNICA – Caries Research Unit, Universidad El Bosque, Bogotá, Colombia; b School of Dental Sciences, Faculty of Medical Sciences, Newcastle University, Newcastle upon Tyne, United Kingdom; c Newcastle upon Tyne Hospitals NHS Foundation Trust, Newcastle upon Tyne, United Kingdom; d Grupo de Virología, Universidad El Bosque, Bogotá, Colombia; e Semillero Lún. Grupo D+Tec. Facultad de Ingeniería, Universidad de Ibagué, Ibagué, Colombia

**Keywords:** Bacteriophage, Aerosol, Aerosol-generating procedure, Dental setting, Viral aerosols, Airborne transmission, Mitigation

## Abstract

**Aim:**

Although air-cleaning systems (ACS) and high-volume evacuation (HVE) have been shown to reduce dental bioaerosols, few works have looked at viruses. This study aimed to assess the effects of HVE and ACS on dispersion of aerosolised particles and airborne viruses in a simulation model of dental bioaerosols using a detailed and comprehensive sampling approach and different tracer molecules.

**Methods:**

MS2 bacteriophage and fluorescein solution were used as viral or dispersion tracers, respectively. These were added independently to the instrument irrigation system and aerosolised during dental procedures. Aerosol mitigation condition groups were ACS, HVE, ACS plus HVE, and No mitigation (control). Aerosols were collected via settlement onto sterile filter papers and agar plates. In addition, a 6-stage Andersen cascade impactor was used for viral air sampling. Fluorescent particles were analysed using ImageJ software, and bacteriophage was quantified using plaque assays and reverse transcription quantitative polymerase chain reaction (RT-qPCR).

**Results:**

HVE combined with ACS produced the highest reduction in viral spread among the three aerosol-generating procedures (AGPs). This reduction was evident both in the number of fluorescent particles and that of viruses. The effect was more pronounced at the farthest sites. By plaque assay, viral loads in the largest-sized aerosols (>7.0 μm) from air samples were 93% lower in the ACS plus HVE group compared with control, and the load in the smallest aerosols (0.65-1.1 μm) was reduced by 84% (*P* < .05). By RT-qPCR, the ACS plus HVE group also reduced viral detection in air samples (>80%) more than ACS or HVE alone compared with the control group (*P* < .05). In ACS or HVE condition, viral RNA was detected even in the absence of infectious virus detected by plaque assay for anterior versus posterior procedures and in the farthest positions.

**Conclusions:**

A synergistic effect was obtained in the reduction of aerosolised particles and viruses from surfaces and air samples when HVE was combined with ACS.

**Clinical Relevance:**

Using a comprehensive and depth assessment, this study found that the combination of high-volume evacuation and air-cleaning systems was the most effective measure in mitigating virus dispersion during aerosol-generating dental procedures, translated into lower required waiting (fallow) times between patients’ appointments for dental care depending on the type of AGP conducted. The more aerosols are produced during an AGP, the longer it takes to prevent cross-contamination among dental staff and patients. In addition, our findings support the use of HVE, face shields, and well-fitting masks in dental procedures, not only during pandemics but also in everyday practice.

## Introduction

Aerosol-generating dental procedures (AGPs) can produce significant amounts of potentially infectious droplets and aerosols.^1^, ^2^, ^3^ Although several dental studies have investigated aerosol dispersion, only a few have employed viral tracers to assess mitigation strategies aimed at reducing infection risk in dental settings.^3^, ^4^, ^5^, ^6^

The exploration of viral dispersion in dental practices has been limited, primarily because of the challenges associated with studying human viruses in indoor environments. Bacteriophages, such as bacteriophage MS2 (*Emesvirus zinderi*), have been proposed as valuable non-pathogenic surrogates for viruses relevant to human health.^7^ MS2 belongs to the non-enveloped *Leviviridae* family and features a capsid approximately 25 nm in diameter, containing a single-stranded ribonucleic acid (ssRNA) genome composed of 3,569 nucleotides.^8^ Methods for collecting viral aerosols include both settlement and active air-sampling techniques.^4^^,^^8^ One notable active sampling method is the Andersen impactor,^9^ which has been used to capture microorganisms including bacteria and endotoxin present in aerosol particles generated during simulated dental procedures.^10^ This active air sampler (flow rate of 28.3 L/min) simulates human inhalation by collecting particles in 6 stages according to their aerodynamic diameter: >7.0 μm, 4.7-7.0 μm, 3.3-4.7 μm, 2.1-3.3 μm, 1.1-2.1 μm and 0.65-1.1 μm^11^. As far as we know, just one paper has looked at the use of bacteriophages and the Anderson impactor to mimic the entrance and circulation of viral particles through the airway compartments based on their aerodynamic size in dental scenarios.^6^

Increasing the room air ventilation rate within dental treatment rooms has been proposed as a viable strategy to reduce the risk of indoor infections, and where this is not possible, using air filtration to improve the effective ventilation rate.^12^^,^^13^ In this context, the American Society of Heating, Refrigeration and Air Conditioning Engineers (ASHRAE) has suggested the use of air purification devices (such as air cleaner systems (ACS)) to reduce aerosol of different sizes.^14^^,^^15^ Using such devices as close to the patient as feasible without impeding air intake and discharge, the air is passed through high-efficiency particulate air (HEPA) filters to remove particulates, and the clean air then returned to the clinical area. Whether air cleaners can affect viral aerosols in the dental practice remains unclear.^14^^,^^15^

On the other hand, high-volume evacuation (HVE, also described as suction or aspiration in the literature) uses a suction tip operating at >300 L/min placed into the mouth to capture liquid, aerosols and droplets produced by a dental procedure. Balanta-Melo et al. found that HVE could reduce generated particles but not eliminate them.^16^ Therefore, HVE has been recommended in dental clinical practice guidelines during the COVID-19 pandemic as a mitigation measure.^16^, ^17^, ^18^, ^19^ However, studies examining HVE have faced limitations, including a low number of repetitions of AGPs and sampling sites. In addition, the gradual emergence of new strains of viruses historically has led to unexpected and unprecedented global challenges such as pandemics.^20^ Unfortunately, it is highly probable that additional pandemics will occur in the future. This highlights the need for scaling up sustainable and effective early warning systems to detect viral hazards, as well as the assessment of mitigation strategies to counteract infections during health emergency situations (e.g. SARS, Influenza, COVID-19).^21^^,^^22^

As far as we know, there is not enough evidence around aerosols mitigation using a comprehensive and depth assessment merging different tracers and collection methods. Therefore, the objective of the present study was to assess the effectiveness of HVE and ACS, both individually and in combination, in mitigating virus dispersion in aerosols during dental procedures.

## Materials and methods

### Bacterial culture and bacteriophage propagation

MS2 bacteriophage was sourced from the Félix d’Hérelle Reference Center for Bacterial Viruses at Université Laval in Québec, Canada, and propagated in trypticase soy broth (BD BBL Trypticase Soy Broth (Soybean-Casein Digest Medium)), using its host bacteria, *Escherichia coli* (Migula Castellani and Chalmers 15597-B1). For propagation, the bacteriophage and bacterial host cells were incubated in 10 mL of TSB media for 18 hours at 25 °C, and centrifuged at 150 RPM (Forma Scientific). The bacteriophage was added when OD_600nm_ reached 0.1. Cells and debris were removed from the phage lysate by centrifugation at 3,500 RPM for 10 min at room temperature. Bacteriophage-containing supernatant was filtered (0.45 µm) and then kept at 4 °C until use. Supernatants were titrated using a conventional double-layer plating assay, which served as the stock solution. Tenfold dilutions of the phage stock prepared in TSB were mixed with an overnight culture of *Escherichia coli* and soft agar (TSB supplemented with 0.7% agar). The mixtures were then poured onto the surface of TSB plates (1.5% agar) and incubated overnight at 25 °C.

### Setting and simulation conditions

A non-controlled ventilated 27 m^3^ (3 × 3 × 3 m (l x h x w)) single-unit dental setting was adapted for the study. Experiments were conducted using a dental mannequin (Bader No. 26810) that simulated the head and oral cavity. The mannequin was equipped with a dental model featuring 28 removable artificial teeth adapted to gums located on adjustable metal plates (Typodont OM860). Polyvinyl siloxane silicone (Lab-putty, Coltene/Whaledent) was used to recreate the normal dimensions of the oral cavity. For anterior or posterior teeth procedures during AGPs, natural human anterior and posterior teeth obtained from the tooth bank at UNICA – Caries Research Unit at Universidad El Bosque (previously stored in 0.002% thymol) replaced the artificial teeth (ethical approval PCI-2016-8803).

Fluorescein solution (2.65 mM) was used as an overall aerosol dispersion tracer, and suspensions of bacteriophage MS2 (approximately 1 × 10^8^ plaque-forming units (PFU)/mL) were used as a viral aerosol dispersion tracer. Each tracer was independently added to the water reservoir of the dental unit to be used, in separate experiments.

The experimental setup aimed to mimic closely a real-world dental setting while allowing for controlled conditions and the use of tracers to monitor aerosol and viral dispersion during dental procedures. Fluorescent solution or bacteriophage MS2 was independently introduced into the water tank that supplied irrigation for the dental instruments, facilitating aerosolisation during the AGPs.

### Dental procedure and aerosol control measures

**Fig. 1:**
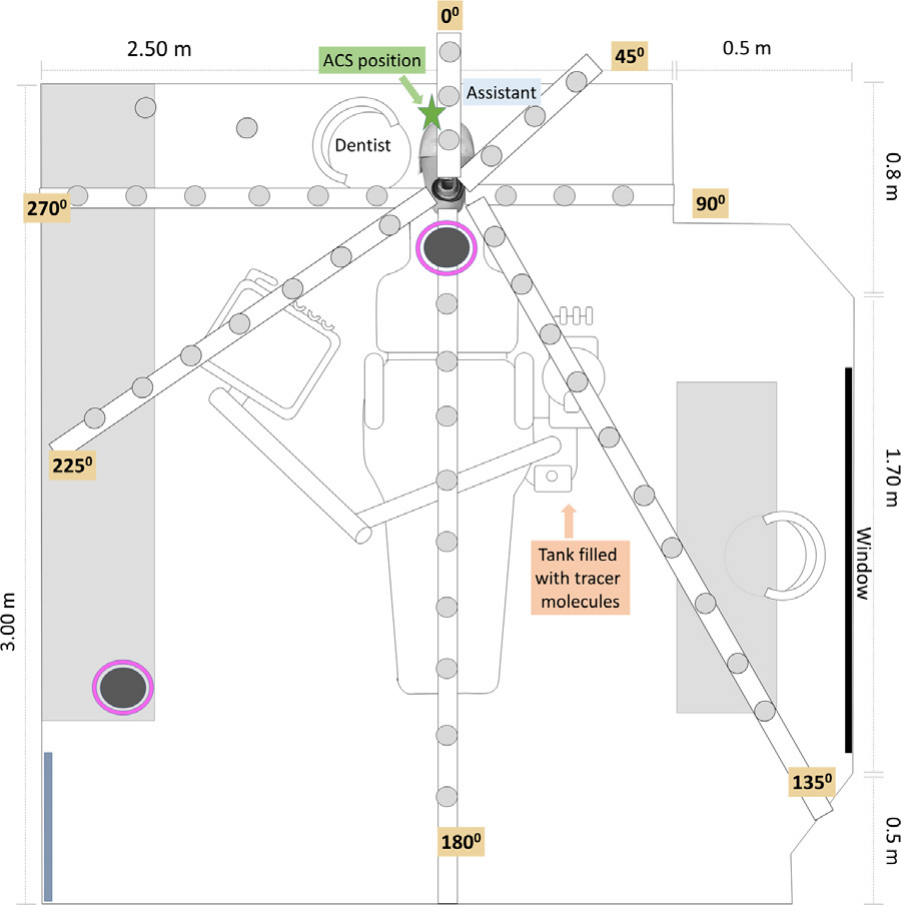
Schematic representation of the dental office design and adaptation. Aerosol collection sites by sedimentation (light grey circles) or impaction using the 6-stage Andersen cascade impactor (dark grey circles, pink bordered).

The procedures included posterior and anterior teeth composite restorations (3M Filtec 350XT One Universal), which involved class II and IV Black's cavity preparations on natural teeth, entailing the removal of dental tissue from at least 2 tooth surfaces.^23^ A high-speed air turbine handpiece (450,000 RPM, KAVO; irrigant flow rate: 34.31 mL/min) and round diamond burs (JOTA) were used, followed by the filling of cavities with composite materials (Filtek Z350-3MTM).^23^ Additionally, water and air from a triple syringe were used during the procedures to wash and dry the work area. During full-mouth oral hygiene procedures, an ultrasonic scaler was activated (Kavo; Frequency: 6,000 Hz; irrigant flow rate: 50 mL/min). All AGPs were conducted by a trained dentist, assisted by a dental nurse (Figure 1) who was in charge of providing dental instruments and materials as well as support for suction and aerosol mitigation strategies activation. The duration of each AGP is detailed in Appendix 1. All procedures were conducted in triplicate under the following conditions: (1) control, utilizing only low-volume aspiration (suction rate: < 280 L/min); (2) mitigation employing an air-cleaning system (ACS) (KEENPURE; equivalent air change rate per hour: 6; reported clean air delivery rate ≈ 195 cubic feet per minute (CFM) in conjunction with low-volume aspiration (LVA)); (3) high-volume aspiration (HVE; suction rate: 280-300 L/min); and (4) a combination of ACS and HVE.

### Aerosol collection and measurement

Aerosols generated during and after each AGP were captured independently using cotton-cellulose paper filters (Whatman) for fluorescein or petri dishes with *E. coli* cultures for viral aerosols, positioned at intervals of 30 cm from the mannequin’s mouth (the aerosol-generation source). An adjustable attachment with plastic extensions was used, extending up to 3 m (depending on the space availability) and corresponding to angles of 0°, 45°, 90°, 135°, 180°, 225°, 270° and 315° clockwise from the AGP source (Figure 1). Samples were collected at different times (*t*), from *t*0 to *t*8. *t*0 corresponded to the background collection exposing filters/petri dishes in each position before the first procedure at the beginning of each session for 10 minutes. After conducting the whole procedure and an additional fallow time of 15 min, the next group of samples was collected (*t*1; 0-15 min). Immediately after, new sets of filters or petri dishes with *E. coli* cultures were positioned around the mannequin and replaced from 20 to 180 min as follows: *t*2 (20-35 min), *t*3 (40-55 min), *t*4 (60-75 min), *t*5 (80-95 min), *t*6 (100-115 min), *t*7 (120-150 min) and *t*8 (155-180 min). At *t*1 additional collection filters/plates were placed on the operator's and assistant’s right shoulder, chest, facial visor and N95 mask (*n* = 4 total procedure).

Collected filter papers were positioned between a glass slide and a coverslip, and photomicrographs were captured using a fluorescence lamp and a 5X magnification lens on a fluorescence microscope (Zeiss AXIO Imager.M2). Image analysis was conducted using ImageJ (version 1.53b). The images were converted into 8-bit pixel-scale fractions, allowing for comprehensive analysis across the sample. A manual threshold was established to select areas of varying intensity, facilitating the identification of particles ranging from 0 to infinity per mm² within a defined area. The analysis yielded data on the number of particles, total surface area and average particle size. All data were recorded in an Excel spreadsheet (Windows 365). The number of particles at each collection position served as an indicator of contamination levels in the samples. An independent examiner was trained prior to the formal analysis to ensure consistency and accuracy.

Additionally, the viral infectious load in aerosols generated during each AGP was assessed using host bacteria seeded in petri dishes that were exposed during and after each procedure at each collection site, as shown in Figure 1. For this assessment, a double-layer agar technique was used.^24^ Active air sampling was also performed using the 6-stage Andersen cascade impactor (ACI), which mimicked the compartments of the human respiratory tract during inhalation. The impactor was positioned 30 cm apart from the mannequin's mouth (Figure 1) and activated at a flow rate of 28.4 L/min during the AGP. An additional activation of the impactor was conducted at a distance of 1.5 m at a 225° angle from *t*2 to *t*3. Regardless of the sampling method used (settling or impaction), the collected culture plates were incubated at 37 °C for 18 hours. At this time, PFUs, which serve as indicators of viable infective virus, were counted.

Following incubation, all collected plates (both containing PFUs and without) were washed with SM buffer (10 mM Tris–HCl, 100 mM NaCl, 10 mM MgSO_4_, adjusted to pH 7.4) while being agitated on a shaker (250 RPM) for 1 hour to recover MS2. The washing solution was then recovered. For quantification of MS2, genomic RNA was extracted using a QIAamp viral RNA minikit following the manufacturer’s instructions. Briefly, the RNA carrier was omitted from the Qiagen AVL buffer, and the RNA was eluted from the column with 40μl TE buffer, pH 8.0 (10 mM Tris, 0.1 mM EDTA). All RNA samples were stored at –80 °C. MS2 cDNA synthesis was performed using an iScript cDNA synthesis kit.

The primers and probes used in this study are described as follows: MS2F: 5 ´-GTCCATACCTTAGATGCGTTAGC-3 ´; MS2R: 5 ´-CCGTTAGCGAAGTTGCTTGG-3 ´. The probes were labelled with 6-carboxyfluorescein (FAM) at the 5 ´ end and Iowa black FQ (IABlkFQ) or black hole quencher 1 (BHQ) at the 3 ´ end (5 ´ -FAM-ACGTCGCCAGTTCCGCCATTGTCG-BHQ-3 ´). The assay components per 20 μl were 5 μl of each sample, 12.5 pmol of primers and 12.5 μl of 2X master mix of the iQ Supermix, and 5 pmol probe. The PCR program was as follows: 5 min at 94 °C and then 40 amplification cycles including denaturation at 94 °C for 15 seconds, annealing and elongation at 60 °C for 60 seconds, followed by fluorescence measurement.

All samples were analysed using the CFX96 Biorad equipment and software. For each qPCR run, a standard curve was generated in duplicate using MS2 samples as positive control.^7^ MS2 bacteriophage was quantified using a plaque-forming assay. From an initial viral title of 1.2 × 10^8^, serial 10-fold dilutions were used to establish standard curves (10^−9^ to 10^−1^). For each sample of unknown concentration, 2 10-fold dilutions made in duplicate were analysed, and the concentration was determined using standard curves. The background was subtracted using the average over cycle range function of the software. Threshold cycle (C_t_) values were determined automatically with the software. The plotting of C_t_ as a function of the logarithm of the amount of genetic material template gave a straight line. The slope of this graph line gave the PCR efficiency (E) according to the equation E = (10^−1/slope^ – 1) × 100. Results were considered accurate when E was over 85%, and the error between standard points and the regression curve was lower than 0.1.

### Statistical analysis

Results obtained in triplicate from each AGP experiment (fluorescence particles, PFUs or RT-qPCR) were analysed using Shapiro–Wilk tests to assess normality. Data were summarised using descriptive analyses. Group differences among the different conditions (Control / ACS / HVE / ACS + HVE) and times were conducted using Mann–Whitney U tests or Kruskal–Wallis Test for multiple comparisons, and a minimum value of *P* < .05 was considered statistically significant.

## Results

Conditions included for the final analyses consisted of anterior, posterior and scaler AGPs and were conducted in triplicate (control: *n* = 9; 3 each). In addition, each AGP was conducted in triplicate using ACS, HVE or ACS plus HVE (*n* = 27; 3 each). Thus, 36 individual experiments using a fluorescent marker and the same number of experiments were conducted using a viral tracer.

### Fluorescent aerosols collection

A total of 344 samples were analysed in each procedure, comprising 43 settlement surfaces in each position at *t*1 (Figure 1), 301 replacements (*t*2-*t*8), plus 5 collection media set on the practitioner and 5 on the assistant in each procedure (Table 1).

**Table 1:** Droplets/Aerosol Settlement on the Practitioner and Assistant Measured by Contaminated Surface Area Using Image Analysis in t1.

		Dentist				Assistant			
	Fluorescent particles number	Right hand	Chest	Respirator	Face-shield	Right hand	Chest	Respirator	Face-shield
AGP	Posterior	16,209 ± 324	15,658 ± 235	89 ± 24	1,967 ± 135	16,856 ± 235	43 ± 9	0 ± 0	684 ± 154
	Anterior	16,856 ± 123	16,587 ± 265	151 ± 5	6,280 ± 279	16,985 ± 231	2,145 ± 68	13 ± 5	1,125 ± 26
	Scaler	15,698 ± 456	16,998 ± 256	164 ± 42	9,637 ± 284	16,354 ± 659	8,272 ± 65	21 ± 12	3,421 ± 325

For each experimental condition, the data from an average of 3 repetitions are shown.

In *t*1the fluorescent solution used as a tracer showed significant dispersion near the aerosol generation area, practitioner and assistant positions. A large number of fluorescent particles were found on the practitioner's hands, chest and on/below the face- shield. Notably, both the forehead and N95 mask (worn under the face-shield) of the practitioner and assistant showed contamination across all AGPs. The lowest number of fluorescent particles on the dental practitioner and assistant were recovered during posterior AGPs compared to anterior teeth instrumentation (*P* < .05; Mann–Whitney U tests) and scaler AGPs (*P* < .01; Mann–Whitney U tests) (Table 1).

Table 2 presents the number of fluorescent particles found at *t*1 (corresponds to particles collected during and 15 min after posterior AGP conduction). Briefly, the highest dispersion was found during scaler AGPs (*P* < 0.01 versus posterior teeth; Mann–Whitney U tests), followed by anterior AGPs (*P* < .05 versus posterior teeth; Mann–Whitney U tests). During scaler and anterior AGPs, contamination was detected in more than 82% of the collection sites. The diameter of fluorescence on filter papers decreased with increasing distance from the aerosol generation source. The maximum collection distance for anterior teeth and scaling procedures was 180 cm at 180°, whereas for posterior AGPs it was 90 cm at 180°.

**Table 2:** Fluorescence Particles Number at *t*1 .

Angle Time (min)		0	45	90	135	180	225	270	315
Posterior AGP	G30	1,587 ± 516	14,653 ± 1,301	12,975 ± 849	17,367 ± 944	14,110 ± 181	15,195 ± 230	13,493 ± 527	N.D
	60	3,012 ± 934	0 ± 0	16,906 ± 1,385	14,981 ± 463	13,706 ± 518	10,663 ± 556	13,889 ± 452	N.D
	90	1,920 ± 78	147,72 ± 401	12,181 ± 894	0 ± 0	12,853 ± 611	14,004 ± 557	16,091 ± 688	0 ± 0
	120	N.D	0 ± 0	0 ± 0	0 ± 0	0 ± 0	0 ± 0	0 ± 0	0 ± 0
	150	N.D	N.D	N.D	0 ± 0	0 ± 0	0 ± 0	0 ± 0	N.D
	>150	N.D	N.D	N.D	0 ± 0	0 ± 0	0 ± 0	0 ± 0	N.D
Posterior AGP with ACS	30	1,322 ± 153	12,746 ± 315	12,125 ± 311	12,590 ± 746	13,680 ± 517	10,700 ± 320	10,075 ± 290	N.D
	60	1,476 ± 59	0 ± 0	13,659 ± 1,715	10,226 ± 118	12,280 ± 59	11,647 ± 1,047	10,031 ± 149	N.D
	90	336 ± 108	12,275 ± 699	14,888 ± 268	10,611 ± 780	12,527 ± 455	12,191 ± 900	10,643 ± 406	0 ± 0
	120	N.D	0 ± 0	0 ± 0	0 ± 0	0 ± 0	0 ± 0	0 ± 0	0 ± 0
	150	N.D	N.D	N.D	0 ± 0	0 ± 0	0 ± 0	0 ± 0	N.D
	>150	N.D	N.D	N.D	0 ± 0	0 ± 0	0 ± 0	0 ± 0	N.D
Posterior AGP with HVE	30	3,189 ± 279	3,992 ± 237	4,589 ± 1,689	6,187 ± 647	6,916 ± 209	4,985 ± 135	6,952 ± 793	N.D
	60	4,145 ± 98	6,723 ± 706	7,029 ± 460	3,961 ± 180	4,255 ± 348	4,245 ± 267	6,605 ± 458	N.D
	90	4,654 ± 564	4,111 ± 290	6,222 ± 441	4,030 ± 92	4,133 ± 149	2,887 ± 132	7,142 ± 784	0 ± 0
	120	N.D	0 ± 0	0 ± 0	0 ± 0	0 ± 0	0 ± 0	0 ± 0	0 ± 0
	150	N.D	N.D	N.D	0 ± 0	0 ± 0	0 ± 0	0 ± 0	N.D
	>150	N.D	N.D	N.D	0 ± 0	0 ± 0	0 ± 0	0 ± 0	N.D
Posterior AGP + HVE + ACS	30	926 ± 47	1,339 ± 84	1,134 ± 71	1,342 ± 81	3,578 ± 1397	1,134 ± 106	884 ± 102	N.D
	60	882 ± 88	0 ± 0	4,904 ± 1,164	2,795 ± 297	3,660 ± 2,239	1,246 ± 92	942 ± 70	N.D
	90	1,235 ± 108	1,032 ± 120	1,370 ± 225	1,065 ± 29	1,062 ± 135	1,149 ± 149	3,253 ± 1,600	0 ± 0
	120	N.D	0 ± 0	0 ± 0	0 ± 0	0 ± 0	0 ± 0	0 ± 0	0 ± 0
	150	N.D	N.D	N.D	0 ± 0	0 ± 0	0 ± 0	0 ± 0	N.D
	>150	N.D	N.D	N.D	0 ± 0	0 ± 0	0 ± 0	0 ± 0	N.D
Anterior AGP	30	12,820 ± 416	12,626 ± 365	11,637 ± 191	13,394 ± 449	14,582 ± 488	12,450 ± 153	12,994 ± 223	N.D
	60	16,344 ± 933	11,769 ± 413	11,827 ± 256	147,09 ± 491	14,513 ± 2152	14,056 ± 775	14,201 ± 1,038	N.D
	90	13,160 ± 212	12,409 ± 534	10,073 ± 134	12,191 ± 151	12,676 ± 327	12,054 ± 239	11,595 ± 523	16,989 ± 845
	120	N.D	0 ± 0	0 ± 0	0 ± 0	8,589 ± 316	0 ± 0	0 ± 0	13,927 ± 3,031
	150	N.D	N.D	N.D	0 ± 0	3,453 ± 50	0 ± 0	0 ± 0	0 ± 0
	>150	N.D	N.D	N.D	0 ± 0	0 ± 0	0 ± 0	0 ± 0	N.D
Anterior AGP with ACS	30	12,055 ± 607	12,940 ± 132	11,668 ± 591	12,528 ± 441	13,533 ± 444	10,861 ± 163	12,078 ± 593	N.D
	60	12,528 ± 64	12,765 ± 312	11,281 ± 287	12,064 ± 1714	12,386 ± 164	12,569 ± 262	11,157 ± 931	N.D
	90	12,825 ± 691	11,147 ± 359	11,775 ± 233	107,78 ± 2,015	11,061 ± 123	10,531 ± 357	10,807 ± 1,512	16,340 ± 1,166
	120	N.D	0 ± 0	0 ± 0	0 ± 0	11,758 ± 404	0 ± 0	1,676 ± 188	14,331 ± 1,162
	150	N.D	N.D	N.D	0 ± 0	1,859 ± 1,200	0 ± 0	0 ± 0	N.D
	>150	N.D	N.D	N.D	0 ± 0	0 ± 0	0 ± 0	0 ± 0	N.D
Anterior AGP with HVE	30	6,209 ± 826	5,400 ± 959	9,333 ± 777	5,758 ± 653	7,454 ± 139	6,222 ± 1,636	8,720 ± 743	N.D
	60	6,605 ± 1,736	3,909 ± 532	7,140 ± 1,016	3,858 ± 725	3,039 ± 534	3,619 ± 1,377	6,330 ± 112	N.D
	90	5,950 ± 411	4,147 ± 916	5,339 ± 764	3,064 ± 100	2,240 ± 195	3,189 ± 661	7,273 ± 654	0 ± 0
	120	N.D	0 ± 0	0 ± 0	0 ± 0	460 ± 215	0 ± 0	0 ± 0	0 ± 0
	150	N.D	N.D	N.D	0 ± 0	0 ± 0	0 ± 0	0 ± 0	N.D
	>150	N.D	N.D	N.D	0 ± 0	0 ± 0	0 ± 0	0 ± 0	N.D
Anterior AGP + HVE + ACS	30	4,233 ± 148	2,426 ± 107	3,059 ± 294	2,308 ± 183	2,962 ± 76	2,224 ± 99	6,287 ± 534	N.D
	60	4,221 ± 88	1,169 ± 108	3,323 ± 89	2,714 ± 203	4,205 ± 164	2,931 ± 55	5,832 ± 1,296	N.D
	90	4,096 ± 1,145	1,976 ± 281	3,494 ± 150	1,806 ± 289	2,236 ± 69	1,173 ± 208	5,357 ± 88	0 ± 0
	120	N.D	0 ± 0	0 ± 0	0 ± 0	0 ± 0	0 ± 0	0 ± 0	0 ± 0
	150	N.D	N.D	N.D	0 ± 0	0 ± 0	0 ± 0	0 ± 0	N.D
	>150	N.D	N.D	N.D	0 ± 0	0 ± 0	0 ± 0	0 ± 0	N.D
Scaler AGP	30	1,578 ± 231	13,348 ± 529	61,323 ± 718	14,080 ± 980	14,309 ± 333	14,824 ± 305	12,575 ± 352	N.D
	60	12,669 ± 531	13,352 ± 596	14,755 ± 268	17,837 ± 1,007	12,779 ± 726	1,396 ± 45	11,040 ± 837	N.D
	90	13,760 ± 627	12,142 ± 974	13,648 ± 394	9,734 ± 496	14,062 ± 925	170,43 ± 775	13,684 ± 562	16,414 ± 1479
	120	N.D	0 ± 0	0 ± 0	0 ± 0	14,955 ± 1,317	0 ± 0	12,392 ± 220	16,957 ± 1,005
	150	N.D	N.D	N.D	0 ± 0	0 ± 0	0 ± 0	11,593 ± 2,049	N.D
	>150	N.D	N.D	N.D	0 ± 0	0 ± 0	0 ± 0	0 ± 0	N.D
Scaler AGP + ACS	30	7,369 ± 82	106,27 ± 578	200,01 ± 3918	11,397 ± 501	10,827 ± 418	10,877 ± 454	12,410 ± 120	N.D
	60	10,952 ± 516	10,597 ± 525	14,003 ± 930	13,232 ± 929	12,211 ± 1019	1,376 ± 74	11,102 ± 215	N.D
	90	12,454 ± 1,407	10,197 ± 650	11,839 ± 552	8,000 ± 713	12,663 ± 538	14,321 ± 813	13,193 ± 1494	14,826 ± 282
	120	N.D	0 ± 0	0 ± 0	0 ± 0	12,985 ± 807	0 ± 0	10,939 ± 512	14,938 ± 553
	150	N.D	N.D	N.D	0 ± 0	0 ± 0	0 ± 0	11,682 ± 543	N.D
	>150	N.D	N.D	N.D	0 ± 0	0 ± 0	0 ± 0	0 ± 0	N.D
Scaler AGP + HVE	30	985 ± 95	7,293 ± 2,080	4,684 ± 389	9,959 ± 1,222	7,075 ± 1,056	5,862 ± 426	11,224 ± 1037	N.D
	60	4,955 ± 873	8,488 ± 533	4,874 ± 323	42,924 ± 52,442	8,182 ± 2,893	473 ± 151	8,533 ± 1,608	N.D
	90	5,352 ± 1,620	3,223 ± 1,496	4,418 ± 588	8,705 ± 729	4,943 ± 1,064	5,151 ± 1,202	10,661 ± 497	0 ± 0
	120	N.D	0 ± 0	0 ± 0	0 ± 0	7,525 ± 1,049	0 ± 0	0 ± 0	0 ± 0
	150	N.D	N.D	N.D	0 ± 0	0 ± 0	0 ± 0	0 ± 0	N.D
	>150	N.D	N.D	N.D	0 ± 0	0 ± 0	0 ± 0	0 ± 0	N.D
Scaler AGP + HVE + ACS	30	142 ± 23	4,991 ± 1,090	3,252 ± 115	8,035 ± 1,299	2,619 ± 921	1,107 ± 39	1,322 ± 59	N.D
	60	2,894 ± 34	8,225 ± 1,055	3,312 ± 162	3,453 ± 103	92 ± 11	0 ± 0	3,500 ± 541	N.D
	90	1,941 ± 62	6,410 ± 148	1,557 ± 184	176 ± 22	6,296 ± 999	0 ± 0	758 ± 119	0 ± 0
	120	N.D	0 ± 0	0 ± 0	0 ± 0	4,860 ± 1,120	0 ± 0	0 ± 0	0 ± 0
	150	N.D	N.D	N.D	0 ± 0	0 ± 0	0 ± 0	0 ± 0	N.D
	>150	N.D	N.D	N.D	0 ± 0	0 ± 0	0 ± 0	0 ± 0	N.D

Collection corresponds to particles collected during and 15 min after each AGP conduction. Data in each cell shows the average + SD from three replicates in each position (angle/degrees in relation with the AGP source). Positions correspond to those represented in Figure 1.

In *t*1, a predominant particle size of <5 μm was found on the filters in the nearest positions from the aerosol generation source after anterior and scaler AGPs. The latter produced finer particles that sedimented more than in the other procedures (*P* < .005; Kruskal–Wallis Test).

**Fig. 2:**
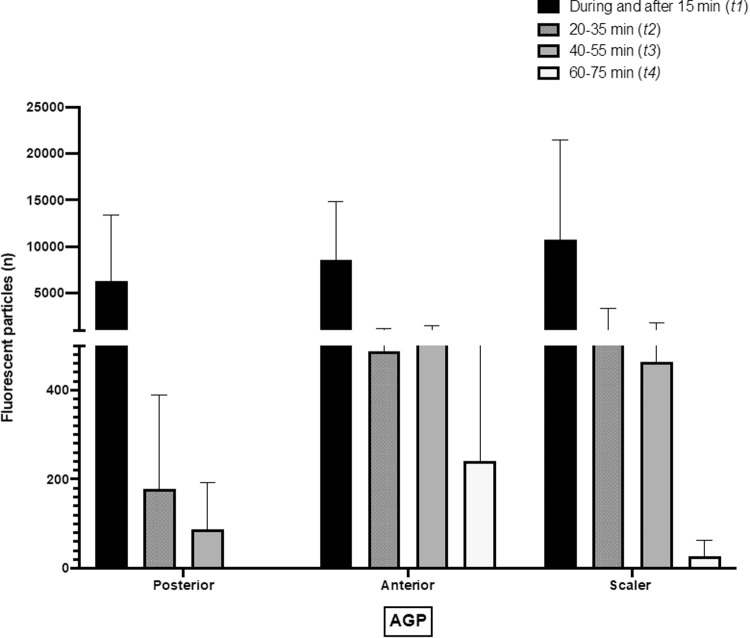
Reduction of aerosolised particles at 4 different evaluation times. Bars show the average of the number of fluorescent particles found in each time from 3 experiments per condition. Error bars show the standard deviation.

Subsequently, from *t2* to *t8*, sedimentation of fluorescent particles was evaluated when the paper filters were replaced, and a reduced number of particles was found for each procedure. Fluorescent particles were detected 40-55 minutes after posterior AGPs and 60-75 minutes (*t4*) after anterior and scaler AGPs (Figure 2). From *t5* to *t8* fluorescent particles were not found on the filter papers.

### The effect of HVE and ACS in mitigation of fluorescent tracer dispersal

**Fig. 3:**
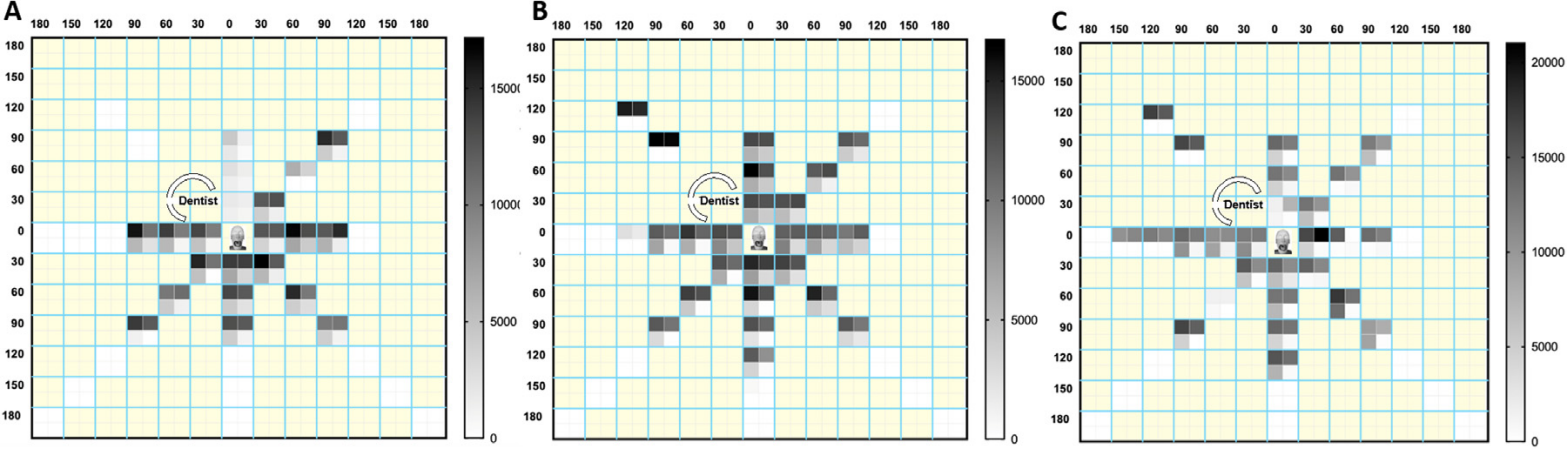
Distribution of fluorescent aerosols during three AGPs (*t*1): (A) Posterior, (B) Anterior and (C) Scaler AGPs. The boxes demarcated by aquamarine blue lines represent each position evaluated. Inside these are the control data (without mitigation) (upper left), ACS (upper right), HVE (lower left), and HVE + ACS (lower right) (Appendix 2). The intensity of the colour represents the greatest number of fluorescent particles as shown in each scale bar.

Figure 3 illustrates the reduction in fluorescent aerosolised particles when using mitigation strategies compared to not using them for each AGP (*t*1). The greatest reduction in aerosol dispersion was observed with HVE compared with ACS, and the most significant reduction occurred when both strategies were combined, compared to the control (AGPs: Posterior: 91.6%; Anterior: 90.1%; Scaler: 90.2%) (*P* < .001).

### Viral aerosols collection

The use of bacteriophage MS2 as a surrogate for respiratory viruses to assess their spread during AGPs revealed different exposure levels for the practitioner and the assistant depending on the procedure. Importantly, the presence of smaller particles was noted even under the face-shield of the practitioner and the assistant after all the AGP conductivity (*t1*) (Posterior: 14 ± 2 PFUs; Anterior 64 *±* 15 PFUs; Scaler 77 *±* 13 PFUs).

**Fig. 4:**
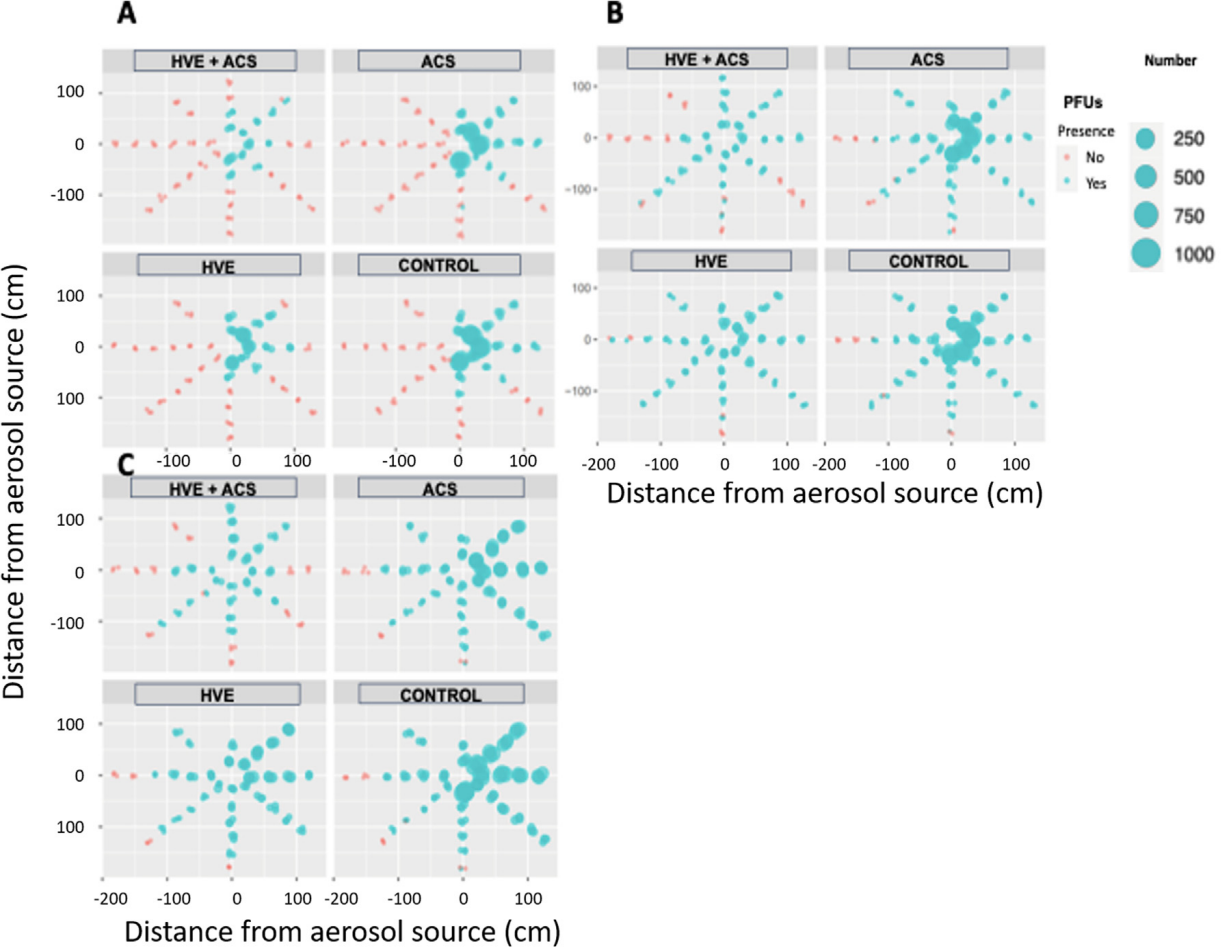
Reduction in PFU counts (mean) obtained from the 3 AGPs through passive settling using each aerosol mitigation strategy and control at t1. (A) Posterior AGP; (B) Anterior AGP; (C) Scaler AGPs. The diameter of the circle at each position is directly proportional to the number of PFUs found.

At *t*1, differential dispersion of viral aerosols was observed among the 3 AGPs by settling. Anterior teeth and scaler AGPs showed greater dispersion of viral aerosols, which were detected at 0° and 315°. During these AGPs, PFUs were found up to *t*3. These approaches allowed the identification of hotspots in each of the AGPs (0°, 90° and 180°) (Figure 4).

### The effect of HVE and ACS in mitigation of viral tracer dispersal

A synergistic effect in reducing aerosols was found among the 3 AGPs when HVE was combined with ACS (Figure 4). This effect was more pronounced at the farthest sites (90-180 cm) for all 3 AGPs. Despite the observed reduction in viral load, no mitigation intervention was able to eliminate the viral load completely. At *t*2 and *t*3, no PFUs were detected after the AGPs when mitigation strategies were used.

### Detection of phage RNA

To complement the overall picture of phage detection, all samples with and without detected PFUs were washed, and the recovered medium was processed using RT-qPCR analysis. As expected, no viral genome amplification was detected from samples used as negative controls (petri dishes with bacteria exposed before each procedure). The highest number of positive samples for all AGPs occurred at *t*1, with lower detection in posterior AGPs (*P* < .05) (Table 3). The use of ACS caused a slight decrease in viral RNA detection, with the greatest reduction achieved when HVE was used alone or in combination with ACS. MS2 RNA was detected in at least 92 % of the samples with previous PFUs detected from anterior and scaler detection at *t*1. From posterior AGPs plates, the viral genome was detected in 84% of samples that had presented PFUs and only in 37% of the samples without previously detected PFUs without mitigation (*P* < .01). When mitigation strategies were used, the MS2 viral genome was not detected in samples from posterior teeth procedures starting at *t*2, and in anterior or scaling procedures it was detected up to *t*3 in 8% of the samples located in the closest positions from the AGP source.

**Table 3:** Changes in Viral Genome Detection (copies/Ml) in 3 Hotspots From 3 AGPs Through Passive Settling Using Each Aerosol Mitigation Strategy and the Control.

**Condition**			**Control**	**ACS**	**HVE**	**HVE + ACS**
**Angle/direction 0-315°**	**AGP Posterior**	Mean	2.03E+04	1.02E+04	1.02E+01	1.02E+01
		Range	E+00 - 1.04E+06	E+00 - 1.01E+06	1.1E+04	2.1E+04
		*P*		>.05	≤.04	≤.04
	**AGP Anterior**	Mean	5.40E+06	1.03E+07	4.01E+05	2.04E+04
		Range	1.83E+03 - 7.47E+09	9.0E+05 - 3.37E09	1.8E+06 - 1.1E+05	3.9E+05 - 2.9E+02
		*P*		<.001	<.001	<.001
	**AGP Scaler**	Mean	2.30E+08	1.03E+07	2.10E+04	2.04E+03
		Range	1.00E+04 - 3.31E+10	1.02E+02 - 3.13E+10	2.2E+06 - 4.12E+05	4.19E+05 - 5.9E+02
		*P*		≤.04	<.001	<.001
**Angle/direction 90°**	**AGP Posterior**	Mean	3.69E+09	1.59E+06	3.90E+02	1.04E+02
		Range	1.00E+4 - 7.06+E10	1.84E+06 - 1.00E+09	1.2E+05 - 1.8E+06	1.0E+05 - 1.1E+06
		*P*		≤.04	<.001	<.001
	**AGP Anterior**	Mean	3.41E+07	2.03E+06	2.30E+02	1.98E+02
		Range	8.4E+05 - 3.2E+06	9.0E+04 - 3.9E+05	1.1E+05 - 1.8E+06	7.1E+02 - 1.6E+04
		*P*		≤.04	≤.04	≤.04
	**AGP Scaler**	Mean	6.72E+08	2.01E+07	5.21E+04	2.3E+03
		Range	2.3E+06 - 4.12E+09	1.4E+06 - 6.2E+09	3.41E+05 - 6.33E+06	3.4E+02 - 4.4E+04
		*P*		>.05	>.01	>.01
**Angle/direction 180°**	**AGP Posterior**	Mean	2.90E+04	ND	ND	ND
		Range	5.4E+02 - 1.6E+05	ND	ND	ND
		*P*				
	**AGP Anterior**	Mean	6.01E+04	4.6E+03	5.1E+02	1.31E+02
		Range	2.3E+02 - 1.8E+05	2.1E+02 - 2.45E+05	3.1E+02 - 1.7E+03	4.1E+02 - 1.2E+04
		*P*		≤.04	≤.04	≤.04
	**AGP Scaler**	Mean	3.6E+04	2.2E+04	1.8E+02	1.6E+02
		Range	8.3E+05 - 4.3E+06	2.2E+05 - 6.41E+06	1.02E+02 - 1.3E+03	1.01E+02 - 2.4E+04
		*P*		>.05	≤.04	≤.04

Figures correspond to the average of data from samples in each angle from *t*1.

### Air collection

Air collection using the Andersen impactor revealed a large amount of infectious viral aerosols in samples collected during each AGP at 30 cm apart from the aerosol generation source, with lower amounts when collected at a greater distance (1.5 m) at an angle of 225°.

**Fig. 5:**
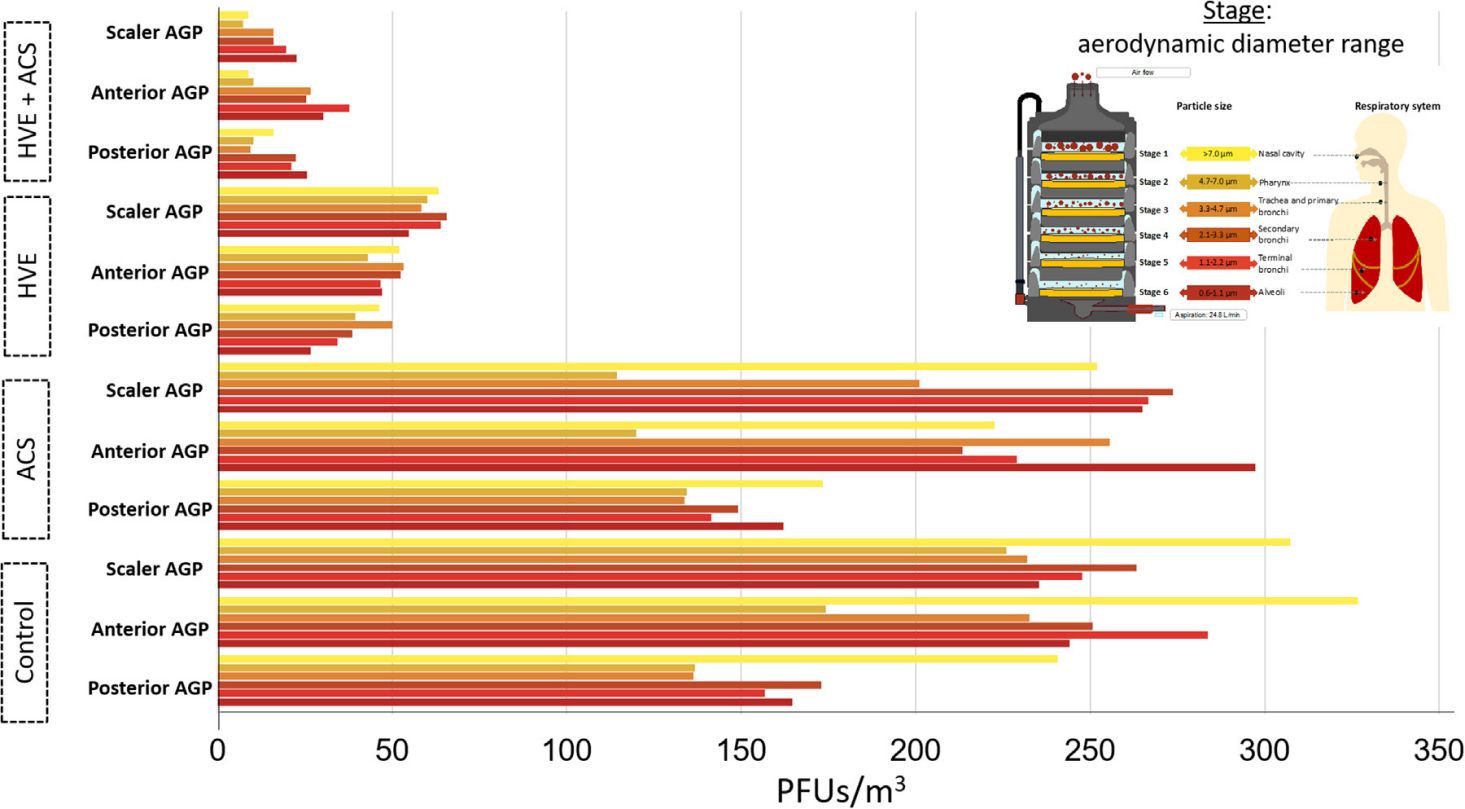
Reduction in PFU counts (median) obtained among AGPs via air sampling using a 6-stage Andersen impactor and different aerosol mitigation strategies and control. This approach revealed the collection of PFUs in all stages (and aerodynamic diameters). Stages 5 (1.1-2.1 µm) and 6 (0.65-1.1 µm) mimic access to the lower respiratory airways.

At *t*1, the use of the ACS slightly reduced the PFU concentration during posterior and anterior AGPs (11.4% and 14.2, respectively), whereas a more significant decrease was observed in scaler AGP (20.2%) (Figure 5). The correspondent figures for ACS plus HVE were Posterior: 89.3%; Anterior: 92.2%; Scaler: 94.8%. The smallest particles (0.65-1.1 μm) persisted mainly during anterior teeth instrumentation and scaling. The effect of using HVE was more evident in reducing aerosolised particles for all three AGPs; however, in the case of the scaler, the number of captured particles was greater. The combination of ACS and HVE induced a reduction in particles but did not completely eliminate them. In this case, anterior AGPs remained at the highest number even when the combined strategies were used (Figure 5).

Appendix 1 AGP conduction times.

Appendix 2 Conventions for fluorescent particle interpretation in each position.

## Discussion

The current study demonstrated significant aerosol control in a dental AGP model by combining high-volume aspiration (HVE) and an air-cleaning system (ACS), using fluorescent and bacteriophage virus tracers to monitor aerosol spreading via settling collection and air sampling (Andersen impactor). The findings of this work are particularly important given the large number of AGPs conducted during dental care and the possible emergence of new viral pathogens causing infectious disease outbreaks, as was seen during the COVID-19 pandemic.^21^^,^^22^

Dental restorative procedures on anterior and posterior teeth were conducted using natural teeth, while full-mouth scaling was performed on artificial teeth. This approach allowed the identification of occupational exposure “hot spots” for the operator or dentist and the assistant, including on the forehead and the N95 respirator located under the face-shield, as well as under the gloves (using fluorescent marker). In addition, dispersion was detected both at the nearest assessed positions from each aerosol generation source (30 cm) and at the farthest positions along the simulated dental setting (>150 cm). This finding emphasises the need for strict adherence to personal protective equipment recommendations and the establishment of modifications, given that potentially infectious viruses could be expelled from patients during routine care or during infectious disease outbreaks.^22^^,^^25^ In addition, traced aerosols in different positions highlight the importance of using effective products in the routine cleaning of reusable dental instruments and surfaces. Regarding this, an interesting study assessed the cleaning efficacy of commonly available cleaning detergent products against organic bioburden on dental instruments. It showed significant variability in cleaning efficacy among test products, particularly when used in ultrasonic cleaners (Optizyme Ultra: 6 mL/L, Asepti Multizyme: 8 mL/L and Getinge Enzymatic Plus: 20 mL/L).^26^ Thus, determinant factors should be considered to achieve an appropriate decontaminant effect. It includes the type of product, concentration, temperature and cleaning duration for maximising soil removal from dental instruments.^26^

Regarding use of fluorescence as aerosol markers, limitations have been reported elsewhere (e.g. potential loss of critical information because of fluorescence degradation, detection sensitivity variations at different locations within the room, among others), advocating the need to use multiple tracking methods in combination in future explorations.^1^^,^^18^^,^^27^, ^28^, ^29^, ^30^ In this study, a manual threshold was established to select areas of varying intensity, facilitating the particles characterisation in size and the elimination of background fluorescence levels. To supplement this information, a viral tracer was used to model the biological aspects of particle spread showing a differential dispersion depending on the procedure and the collection method.

On the other hand, in the current study, high amounts of viral aerosols were found close to the aerosol-generating source (mannequin’s mouth) and even under the practitioner’s face shield, suggesting a potential risk of viral infection considering the particles ability to cross biosafety barriers. Those aspects have been previously described using aerosolised bacteria and endotoxin in dental AGP models,^10^, ^11^, ^12^, ^13^, ^14^, ^15^, ^16^, ^17^, ^18^, ^19^, ^20^, ^21^, ^22^, ^23^, ^24^, ^25^, ^26^, ^27^, ^28^, ^29^, ^30^ but not as extensively using viral approaches. The inoculum of the MS2 phage (10^8^ PFU/mL), which could be considered as a limitation of this study, was similar to the viral load of significant human pathogens such as SARS-CoV-2 found in saliva samples from infected individuals.^31^, ^32^, ^33^ Data obtained from PFUs were complemented by the results from RT-qPCR analysis. The latter enhanced the sensitivity of bacteriophage tracking by allowing the detection of viral genomes even when PFUs were not detected in each position. This approach increases the ability to detect virus dispersion, leading to a better understanding of the virus spread in the dental setting. To date, few research groups have previously used a similar molecular detection approach using other surrogate viruses, including MS2.^3^^,^^4^^,^^6^^,^^13^^,^^34^, ^35^, ^36^ MS2 bacteriophage immersed in SM buffer (to mimic saliva) was widely used as a surrogate to study pathogenic respiratory viruses; however, real-world bioaerosols vary in size and composition. One single bioaerosol particle may be composed of fine or coarse particulate matter, ionic and organic solutes including allergenic material, as well as microorganisms, which results in particles of different aerodynamic sizes independent of the size of their components (bacteria, fungi or viruses). Despite the exhaustive aerosol tracking carried out in this work, additional approaches could complement our findings. Regarding this, an interesting study assessed the spatial flow of liquid droplets through a combination of imaging and numeric data from simulated dental procedures. This novel strategy led to an appropriate characterisation of droplet particle splashing velocity, maximum height and spray angle.^37^ Thus, more effective interventions based on the spatial and temporal distribution of droplets could be designed for public disease prevention and control.^37^

In this study, fluorescent and viral aerosols were substantially reduced in both surface and air samples when HVE or HVE combined with ACS were used. The mechanism of action for HVE appears to be associated with the direct trapping of particles before they are expelled from the oral cavity, resulting in a significant reduction in collected material across all evaluated conditions. A relevant point to mention regarding the reliability of the obtained data is that, to prevent cross-contamination of any of the tracer particles used in this study, we established a fallow time of 3 hours between experiments (after the last sample collection). During this period, windows and doors remained closed, and the circulation of people was avoided. Additionally, new full protective PPE was worn and used before each AGP.

Our results highlight the limitations of conventional methods to mitigate finer aerosols, consistent with findings from previous studies.^5^ A possible explanation could be that large droplets and splashes tend to fall ballistically to the ground or close to the source (1 to 2 m), whereas smaller particles remain suspended. In this case, we found finer particles using the Andersen impactor after some time had elapsed since the AGP was performed. The relevance of detecting viruses in bioaerosols at these impactor stages lies in their potential to penetrate the lungs and alveoli, depending on the aerodynamic size and virus concentration within the particles.^3^^,^^37^^,^^38^ Penetration of bioaerosols progressively deeper into the smaller airways has been shown in animal infection models to correlate with more clinically severe disease.^39^ Engineering control strategies include increasing the number of air exchanges per hour by mechanical ventilation, using HEPA filters, and maintaining negative-pressure rooms to minimise indoor infection risk, and the present study demonstrated the usefulness of combining HVE with an ACS; however, the evidence supporting many of these claims is limited or poorly documented, and these approaches may not be economically sustainable.^40^^,^^41^

In contrast to HVE, the use of an ACS alone showed a limited reduction in tracked aerosols (fluorescent and viral). Regarding HVE, in a published controlled split-mouth clinical trial,^42^ low-volume suction (LVS) had significantly less efficiency than HVE to reduce aerosol contamination during cavity preparation or ultrasound.^18^^,^^19^ On the other hand, in this study, the ACS was located between the dental practitioner and the aerosol source. Some research in medical environments has shown relative efficiency in removing aerosols depending on the particle sizes at different locations.^43^ In addition, Chen et al. observed a reduction in dentist exposition to aerosols using ACS in a single dental setting depending on the airflow dynamics.^44^ Taken together, those results have suggested that the relative position of the ACS in relation to the source of aerosol and the dentist, airflow dynamics and the aerodynamic diameter of droplets/aerosols are key determinants in reducing the exposure of dental practitioners to aerosols. Additional studies might be conducted to delve deeper into this matter, for example assessing the effect of different ACS positioning sites and/or creating directional airflow that pushes droplets away from patients and towards air filtration units.^45^

In the present study, amplification of the viral genome of bacteriophage MS2 from the collected plates was conducted. It allowed us to expand the detection spectrum even in positions where PFUs were not detected. The relevance of these findings relies on the feasibility of those collected viruses remaining viable on surfaces becoming infectious. To our knowledge, a few papers have evaluated and screened phage MS2 using both PFUs and qRT-PCR.^4^, ^5^, ^6^ This is particularly important because, although bacteriophage infection is highly specific, conferred by the affinity between phage and receptors on the bacterial surface, the absence of PFUs in our assays does not imply that the phage is not dispersed. Additional assays showed that despite no initial PFUs being detected, the phage can remain infectious and subsequently infect susceptible bacterial cells, suggesting the same is likely to be true for human viruses.

This study showed that dispersion of droplets and aerosols over time following the dental procedure differs depending on the procedure conducted, with scaler and anterior AGPs generating more numerous and smaller particles than those from posterior AGPs. These results suggest that in an infectious disease outbreak, there may be a need to establish different waiting (fallow) times before the next patient can be treated depending on the procedure. Our results demonstrated that the combination of ACS and HVE most effectively reduced the time aerosols were detected after the procedure.

Even though, simulation studies are widely accepted to assess viral spreading in different scenarios, the current work has several important limitations. First, it did not account for the inherent variability among patients. Since a fixed phantom was used, it does not exactly replicate patient anatomy, orientation during care, movements, salivary flow, the natural antimicrobial properties of saliva and/or breathing inhalation and exhalation. Additionally, the high viral load directly inoculated into the mouth with the fluorescent marker/virus in the bioaerosol spreading model—used to simulate the worst-case scenario during dental AGPs—does not reflect real-world conditions.^46^^,^^47^ The parameters mentioned, as well as the variability of different clinical settings that were not simulated, could potentially overestimate the effects as well as influence the translation of our results. However, these limitations might have contributed to a better understanding of the overall picture of virus-containing aerosol spread and mitigation under the simulated procedures.^46^^,^^47^

Additionally, considering the similar distance of the forehead and eyes from the infection source, it might be inferred that the eyes would also be exposed to similar bioaerosol. These parameters should be included in future studies.

## Conclusion

Within the limitations of this study, HVE appears to provide the strongest benefits for mitigation of the infection risk from aerosolised viruses during dental procedures. The effects of ACS were more limited, although this provided additional benefits when used in combination with HVE. Results from comprehensive sampling methods and the use of different tracer molecules in this study were consistent and comparable with multiple other approaches. Further work is required to determine the infection risks of different viruses in the dental clinical environment.

## Author contributions

*Conceptualisation:* Beltrán, Castellanos, Velandia-Romero, Martignon*Design:* Beltrán, Allison, Jakubovics, Castellanos, Holliday, Velandia-Romero, Martignon*Data acquisition:* Beltrán, Velandia-Romero, Calvo, Martignon*Data analysis and interpretation:* Beltrán, Allison, Jakubovics, Castellanos, Holliday, Velandia-Romero, Calvo, Forero, Martignon*Writting-first draft:* Beltrán, Allison, Jakubovics, Holliday, Velandia-Romero, Calvo, Martignon*Writting-review and editing:* Beltrán, Allison, Jakubovics, Castellanos, Holliday, Velandia-Romero, Calvo, Forero, Martignon

## Conflict of interests

None disclosed.

## Appendix Supplementary materials

Image, application 1

## Footnotes

Supplementary material associated with this article can be found in the online version at doi:10.1016/j.identj.2025.103963.

## References

[1] Allison J.R., Currie C.C., Edwards D.C. (2021). Evaluating aerosol and splatter following dental procedures: addressing new challenges for oral healthcare and rehabilitation. J Oral Rehab.

[2] Zemouri C., Volgenant C.M.C., Buijs M.J. (2020). Dental aerosols: microbial composition and spatial distribution. J Oral Microbiol.

[3] Beltrán E.O., Castellanos J.E., Corredor Z.L. (2023). Tracing ΦX174 bacteriophage spreading during aerosol-generating procedures in a dental clinic. Clin Oral Investig.

[4] Allison J.R., Dowson C., Jakubovics N.S., Nile C.J., Durham J., Holliday R. (2022). Waterline disinfectants reduce dental bioaerosols: a multitracer validation. J Dent Res.

[5] Vernon J.J., Black E.V.I., Dennis T. (2021). Dental mitigation strategies to reduce aerosolization of SARS-CoV-2. J Dent Res.

[6] Malmgren R., Välimaa H., Oksanen L. (2023). High-volume evacuation mitigates viral aerosol spread in dental procedures. Sci Rep.

[7] Turgeon N., Toulouse M.J., Martel B., Moineau S., Duchaine C. (2014). Comparison of five bacteriophages as models for viral aerosol studies. Appl Environ Microbiol.

[8] Verreault D., Moineau S., Duchaine C. (2008). Methods for sampling of airborne viruses. Microbiol Mol Biol Rev.

[9] Andersen A.A. (1958). New sampler for the collection, sizing, and enumeration of viable airborne particles. J Bacteriol.

[10] Duti S., Veillette M., Mériaux A., Lazure L., Barbeau J., Duchaine C. (2007). Aerosolization of mycobacteria and legionellae during dental treatment: low exposure despite dental unit contamination. Environ Microbiol.

[11] King M.D., McFarland A.R. (2012). Use of an Andersen bioaerosol sampler to simultaneously provide culturable particle and culturable organism size distributions. Aerosol Sci Technol.

[12] Gao X., Wei J., Lei H., Xu P., Cowling B.J., Li Y. (2016). Building ventilation as an effective disease intervention strategy in a dense indoor contact network in an ideal city. PLoS One.

[13] Liu Z., Zhang P., Liu H. (2023). Estimating the restraint of SARS-CoV-2 spread using a conventional medical air-cleaning device: based on an experiment in a typical dental clinical setting. Int J Hyg Environ.

[14] ASHRAE. American Society of Heating, Ventilating, and Air- Conditioning Engineers (ASHRAE) COVID-19 (CORONAVIRUS) Preparedness resources. 2020a. https://www.ashrae.org/file%20library/technical%20resources/covid-19/in-room-air-cleaner-guidance-for-reducing-covid-19-in-air-in-your-space-or-room.pdf. Accessed 13 March 2024.

[15] ASHRAE. American Society of Heating, Ventilating, and Air- Conditioning Engineers (ASHRAE) Position Document on Airborne Infectious Diseases. 2020b. https://www.ashrae.org/file%20library/about/position%20documents/pd_-infectious-aerosols-2022_edited-january-2023.pdf. Accessed 13 March 2024.

[16] Balanta-Melo J., Gutiérrez A., Sinisterra G. (2020). Rubber dam isolation and high-volume suction reduce ultrafine dental aerosol particles: an experiment in a simulated patient. Appl Sci.

[17] Chanpong B., Tang M., Rosenczweig A., Lok P., Tang R. (2020). Aerosol-generating procedures and simulated cough in dental anesthesia. Anesth Prog.

[18] Mupparapu M. (2020). Aerosol reduction urgency in post-COVID-19 dental practice. Quintessence Int Berl Ger.

[19] Baldion P.A., Rodríguez H.O., Guerrero C.A., Cruz A.C., Betancourt D.E. (2021). Infection risk prediction model for COVID-19 based on an analysis of the settlement of particles generated during dental procedures in dental clinics. Int J Dent.

[20] Li X., Mak C.M., Wai Ma K., Wong H.M. (2021). How the high-volume evacuation alters the flow-field and particle removal characteristics in the mock-up dental clinic. Build Environ.

[21] Smith G.J.D., Vijaykrishna D., Bahl J. (2009). Origins and evolutionary genomics of the 2009 swine-origin H1N1 influenza A epidemic. Nature.

[22] World Health Organization (2020.). https://www.who.int/emergencies/diseases/novel-coronavirus-2019/technical-guidance.

[23] Hilton T.J. (2013). Fundamentals of Operative Dentistry: A Contemporary Approach.

[24] Kropinski A.M., Mazzocco A., Waddell T.E., Lingohr E., Johnson R.P. (2009). Enumeration of bacteriophages by double agar overlay plaque assay. Methods Mol Biol.

[25] US Department of Labor: Occupational Safety and Health Administration (OSHA). Guidance on preparing workplaces for COVID-19. 2020. https://www.osha.gov/Publications/OSHA3990.pdf. Accessed 22 December 2023.

[26] Seneviratne C.J., Khan S.A., Zachar J., Yang Z., Kiran R., Walsh L.J. (2025). Efficacy of ultrasonic cleaning products with various disinfection chemistries on dental instruments contaminated with bioburden. Int Dent J.

[27] Holliday R., Allison J.R., Currie C.C. (2021). Evaluating contaminated dental aerosol and splatter in an open plan clinic environment: implications for the COVID-19 pandemic. J Dent.

[28] Bentley C.D., Burkhart N.W., Crawford J.J. (1994). Evaluating spatter and aerosol contamination during dental procedures. J Am Dent Assoc.

[29] Kaufmann M., Solderer A., Gubler A., Wegehaupt F.J., Attin T., Schmidlin P.R. (2020). Quantitative measurements of aerosols from air-polishing and ultrasonic devices: (how) can we protect ourselves?. PLoS One.

[30] Gund M., Isack J., Hannig M. (2021). Contamination of surgical mask during aerosol-producing dental treatments. Clin Oral Investig.

[31] Chen W., Qian H., Zhang N., Liu F., Liu L., Li Y. (2022). Extended short-range airborne transmission of respiratory infections. J Hazard Mater.

[32] Dbouk T., Drikakis D. (2020). On coughing and airborne droplet transmission to humans. Phys Fluids.

[33] Leung N.H.L. (2021). Transmissibility and transmission of respiratory viruses. Nat Rev Microbiol.

[34] Ionescu A.C., Brambilla E., Manzoli L., Orsini G., Gentili V., Rizzo R. (2021). Aerosols modification with H_2_O_2_ reduces airborne contamination by dental handpieces. J Oral Microbiol.

[35] Pratt A., Eckermann N., Venugopalan S.R., Uribe L.M., Barlow L., Nonnenmann M. (2023). Evaluation of aerosols in a simulated orthodontic debanding procedure. Sci Rep.

[36] Fidler A., Steyer A., Manevski D., Gašperšič R. (2022). Virus transmission by ultrasonic scaler and its prevention by antiviral agent: an in vitro study. J Periodontol.

[37] Shu H., Yu X., Zhu X. (2024). Visualisation of droplet flow induced by ultrasonic dental cleaning. Int Dent J.

[38] Fröhlich-Nowoisky J., Kampf C.J., Weber B. (2016). Bioaerosols in the Earth system: climate, health, and ecosystem interactions. Atmos Res.

[39] Morgado-Gamero W.B., Mendoza M., Castillo M. (2019). Antibiotic resistance of airborne viable bacteria and size distribution in neonatal intensive care units. Int J Environ Res Publ Health.

[40] Thomas R.J. (2013). Particle size and pathogenicity in the respiratory tract. Virulence.

[41] Saavedra-Trujillo C. (2020). Consenso colombiano de atención, diagnóstico y manejo de la infección por SARS-COV-2/COVID-19 en establecimientos de atención de la salud. Recomendaciones basadas en consenso de expertos e informadas en la evidencia. Infectio.

[42] Robertson C., Clarkson J.E., Aceves-Martins M., Ramsay C.R., Richards D., Colloc T., CoDER Working Group (2022). A review of aerosol generation mitigation in international dental guidance. Int Dent J.

[43] Kumbargere Nagraj S., Eachempati P., Paisi M., Nasser M., Sivaramakrishnan G., Verbeek J.H. (2020). Interventions to reduce contaminated aerosols produced during dental procedures for preventing infectious diseases. Cochrane Database Syst Rev.

[44] Qian H., Li Y., Sun H., Nielsen P.V., Xinghua H., Xiaohong Z. (2010). Particle removal efficiency of the portable HEPA air cleaner in a simulated hospital ward. Build Simul.

[45] Chen C., Zhao B., Cui W., Dong L., An N., Ouyang X. (2010). The effectiveness of an air cleaner in controlling droplet/aerosol particle dispersion emitted from a patient's mouth in the indoor environment of dental clinics. J R Soc Interface.

[46] Yang G., Wang Y., Chung Chan K. (2024). Effectiveness of air cleaner on mitigating the transmission of respiratory disease in a dental clinic environment. Build Simul.

[47] Innes N., Johnson I.G., Al-Yaseen W. (2021). A systematic review of droplet and aerosol generation in dentistry. J Dent.

