# Supplementary material for: Control of Viral Aerosol Dispersion During Simulated Dental Procedures
Source: Int Dent J. 2025 Oct 23;75(6):103963. doi: 10.1016/j.identj.2025.103963 (PMC12593617; doi:10.1016/j.identj.2025.103963)
Supplement: Supplementary file 1 [file mmc1.docx]

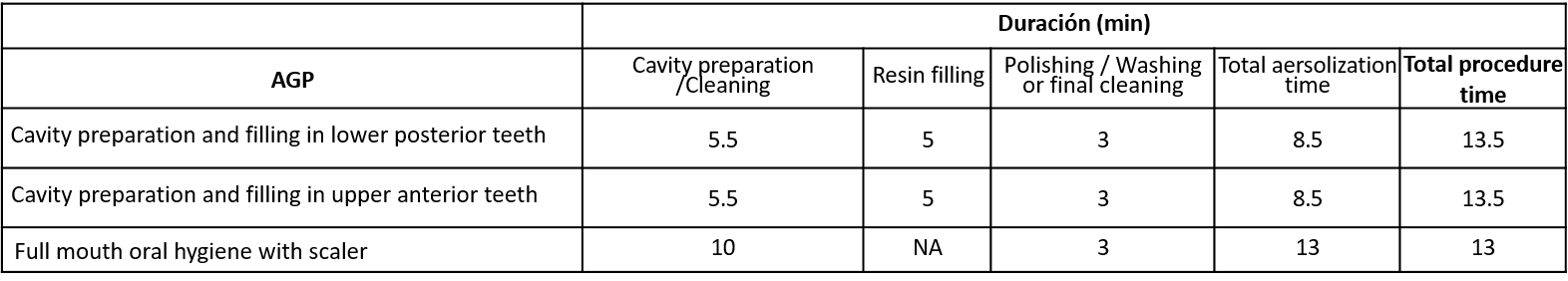


Appendix A. AGP conduction times


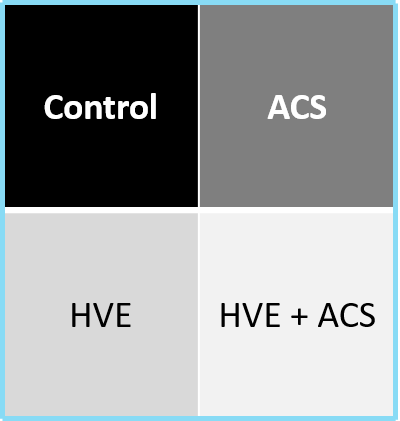


Appendix B. Conventions for fluorescent particles interpretation in each position.
